# Polymorphism in Serotonin Receptor 3B Is Associated with Pain Catastrophizing

**DOI:** 10.1371/journal.pone.0078889

**Published:** 2013-11-11

**Authors:** Emilia Horjales-Araujo, Ditte Demontis, Ellen Kielland Lund, Nanna Brix Finnerup, Anders D. Børglum, Troels Staehelin Jensen, Peter Svensson, Lene Vase

**Affiliations:** 1 Danish Pain Research Center, Aarhus University Hospital, Aarhus, Denmark; 2 Center for Functionally Integrative Neuroscience (CFIN), MindLab, Aarhus University Hospital, Aarhus, Denmark; 3 Department of Biomedicine and Center for Integrative Sequencing (iSEQ), Aarhus University, Aarhus, Denmark; 4 Department of Psychology and Behavioral Sciences, Aarhus University, Aarhus, Denmark; 5 Department of Neurology, Aarhus University Hospital, Aarhus, Denmark; 6 Section of Clinical Oral Physiology, Department of Dentistry, Aarhus University, Aarhus, Denmark; University of Illinois at Chicago, United States of America

## Abstract

Pain catastrophizing, a coping style characterized by excessively negative thoughts and emotions in relation to pain, is one of the psychological factors that most markedly predicts variability in the perception of pain; however, only little is known about the underlying neurobiology. The aim of this study was to test for associations between psychological variables, such as pain catastrophizing, anxiety and depression, and selected polymorphisms in genes related to monoaminergic neurotransmission, in particular serotonin pathway genes. Three hundred seventy-nine healthy participants completed a set of psychological questionnaires: the Pain Catastrophizing Scale (PCS), the State-Trait Anxiety Inventory and Beck’s Depression Inventory, and were genotyped for 15 single nucleotide polymorphisms (SNPs) in nine genes. The SNP rs1176744 located in the serotonin receptor 3_B_ gene (*5-HTR3B*) was found to be associated with pain catastrophizing scores: both the global score and the subscales of magnification and helplessness. This is the first study to show an association between *5-HTR3B* and PCS scores, thus suggesting a role of the serotonin pathway in pain catastrophizing. Since *5-HTR3B* has previously been associated with descending pain modulation pathways, future studies will be of great interest to elucidate the molecular pathways involved in the relation between serotonin, its receptors and pain catastrophizing.

## Introduction

Psychological variables such as pain catastrophizing, anxiety and depression are known to influence the perception of pain [1]–[7]. Recently, both psychological traits and states and pain conditions have been correlated with neurotransmitter systems, and it has been suggested that interindividual differences in the expression of psychological factors and pain perception are based on genetic variations [8]–[10].

Pain catastrophizing along with anxiety and depression is one of the psychological factors that most markedly predict variability in the perception of pain as well as in the development of chronic pain conditions [11]–[13]. Pain catastrophizing has broadly been defined as “an exaggerated negative mental set or orientation brought to bear during actual or anticipated pain experience”, and it is related to attentional bias and hypervigilance [11], [14]. Pain catastrophizing is characterized by a tendency to magnify the threat value of a stimulus by rumination (i.e. a relative inability to inhibit pain-related thoughts) and by a tendency to feel helpless in the context of the stimulus [11], [12], [15]. Conceptually and empirically, pain catastrophizing is closely related to anxiety and depression, [12]. High levels of pain catastrophizing have been associated with increased pain intensity and increased activity in pain-related areas of the brain [16], [17], and also with increased temporal summation [18], [19] and an altered response to opioid treatment [20]. Despite the well-known clinical relevance of pain catastrophizing, the neurobiological underpinnings of the phenomenon have just begun to be specified [13]. So far, only one study has examined possible associations between genetic variation and pain catastrophizing [13].

Serotonin (5-HT) is a neurotransmitter implicated with a wide range of behaviors such as mood and nociception. Serotonergic genes have been extensively studied in relation to pain, anxiety and depression [21]–[25]. However, to date there are no published studies of genetic variations of serotonin-related genes in relation to pain catastrophizing.

The serotonin transporter (5-HTT) plays a critical role in determining the duration and intensity of 5-HT communication with receptors and targets, for review see [26]. The 5-HTT is coded by a single gene (*SLC6A4*) [27]. A 43 base-pair insertion/deletion (referred to as the short “S” and long “L” variant) in the promoter region of the gene in combination with the single nucleotide polymorphism (SNP) rs2553 (A/G) has been shown to alter the degree of gene expression [28], [29]. The combination of these two polymorphisms is referred to as “tri-allelic” 5-HTTLPR and permits a functional division of individuals into “high” (L_A_/L_A_), “intermediate” (L_A_/L_G_; S_A_/L_A_) or “low” (S_A_/S_A_; L_G_/S_A_) expression of the serotonin transporter protein [28].

Expression of low levels of 5-HTT has been associated with neuroticism and anxiety traits [30]–[32]. It has also been suggested that individuals with low expression of the protein have a predisposition for depression [33], eating disorders [34], affective disorders [35] and obsessive-compulsive disorder [36], among others. In addition, Beevers and colleagues, by using a measure of biased attention, were the first to report that carriers of the S-allele showed a bias toward negative stimuli [37], [38]; similar results are also reported in more recent studies [39], [40]. In addition, Fox and colleagues reported strong evidence for a positive bias in the LL group such that vigilance for positive material was observed in addition to clear avoidance of negative material, a pattern that was completely absent in the S-allele carriers [41]. Altogether, these studies suggest that subjects with low 5-HTT expression are more likely to focus on negative stimuli than high expression subjects. Attentional threat bias has been implicated in the etiology and maintenance of anxiety [42]–[44], which strengthens the association between S-carriers and anxiety traits. Thus, it is logic to speculate that S-carriers by focusing on a negative stimulus (e.g. pain) would be more prone to rumination and magnification of such a stimulus and hence to pain catastrophizing.

In recent studies, we [45] and others [46] have found that the tri-allelic polymorphism is correlated with the ability to modulate pain. Thus, participants with the genetic variants associated with high 5-HTT expression experienced increased pain intensity during negative pictures and decreased pain intensity during positive pictures when compared with the pain experienced during neutral pictures. These results were not observed in the genotype group corresponding to the low expression of the protein, probably because of the attentional bias to the painful negative stimuli.

Serotonin neurotransmission might also be affected by polymorphisms in other genes, potentially affecting behavior and personality traits. For instance, it has been suggested that polymorphisms in the serotonin receptor 2_A_ (*5-HT_2A_*) are associated with low anxiety traits and novelty-seeking behavior [47], [48]. Another example is the serotonin receptor 3 (*5-HT_3_*); variations in this gene have been reported to be associated with bipolar disorder [49] and harm-avoidance behavior in women [50]. Moreover, there is now substantial anatomical and functional evidence for the participation of 5-HT3 receptors in spinal nociceptive processing [51]. Since pain catastrophizing is highly associated with processing of noxious stimuli, it is of interest to see if there are associations between genetic variations in these genes and pain catastrophizing [13].

Monoamine oxidase (MAO) is flavin-containing mitochondrial enzymes catalyzing the oxidative deamination of neurotransmitters and biogenic amides in the brain and peripheral tissues [52]. Based on substrate selectivity and inhibitor selectivity, two forms of MAO have been designated: MAO-A and MAO-B [53], [54], which correspond to two distinct genes. Typically, MAO-A catalyzes the oxidation of serotonin (5-HT), whereas MAO-B acts on 2-phenylethylamine and benzylamine [52], [55]. Due to the important role of MAO in monoamine neurotransmission, two SNPs in *MAO-A* and two in *MAO-B* were included in this study.

The aim of this study was to investigate if the psychological factors that most markedly predict pain variability (pain catastrophizing, depression and anxiety) were associated with SNPs in genes related to the serotonin pathway. The SNPs included in this study have previously been reported to be associated with pain perception as well as anxiety and depression [30], [46], [56]–[73].

## Methods

### Participants

As part of a previous study [45], a DNA bank of 380 healthy individuals of Scandinavian descent between 18 and 39 years of age were recruited at Aarhus University. Individuals were excluded from the study if they had any chronic pain condition (based on the IASP definition of chronic pain), were smokers, pregnant, used medication on a regular basis (except for contraceptives), or if they had any known psychological, cardiovascular or neurological disorder.

All participants gave written informed consent upon having received detailed information on the study and received a bottle of wine in compensation for their participation. The study was conducted according to the Declaration of Helsinki and was approved by the local ethical committee (20110165) and the Danish Data Protection Agency (2011-41-6562).

### Study Design

Upon entry in the study, participants completed the following psychological questionnaires: the Pain Catastrophizing Scale (PCS), Beck Depression Inventory (BDI) and the Spielberger State-Trait Anxiety Inventory I and II (STAI), using SurveyXact (Rambøll Management Consulting, Denmark). Afterwards the participants were asked to give a saliva sample.

### DNA Analysis

DNA was extracted from saliva collected using an OC-100 kit (DNA Genotek Inc, Ontario, Canada). To determine the triallelic 5-HTTLPR genotype, PCR reactions were carried out in a total volume of 25 µl using the GoTaq® Hot Start Polymerase (Promega, Wisconsin, USA) and 80 ng of genomic template. The forward primer sequence was 5′-CTCTGAATGCCAGCACCTAACCC-3′ and the reverse 5′-GATTCTGGTGCCACCTAGACGC-3′. Samples were amplified (Gene Amp, PCR System 9700, Applied Biosystems, California, USA) by 2-step PCR consisting of an activation step of 2 min at 94°C, followed by 35 cycles of 30 s denaturation at 93°C, and an annealing and elongation step for 1 min at 62°C, followed by a final elongation step of 10 min at 72°C. The L-allele and the S-allele of the 5-HTTLPR yield a product of 529 bp and 486 bp, respectively.

Fragments were visualized with UV after 45 min of separation at 80 V on a 2.5% agarose gel. In order to ensure that the primers amplified the right DNA region, two random samples were selected, and the PCR product was purified (Jet Quick PCR product purification, Genomed, Löhne, Germany) and Sanger sequenced by Eurofins MWG Operon (Ebersberg, Germany).

To determine the rs25531 genotype, 10 µl of the PCR product was digested for 2 h at 37°C with 1 µl MSP1 (New England Biolabs, Ipswich, MA, USA) and 1 µl buffer per sample. The enzyme cuts at a 5′-C/CGC-3′sequence, resulting in fragments of different lengths, which determined the triallelic genotype (see Table 1). The digested fragments were visualized by UV light after 2 h of separation at 100 V on a 4% agarose gel.

**Table 1:** Genetic polymorphisms genotyped in this study.

Polymorphism (dbSNPdatabase number)	Gene	Physical Position	Chromosome	Position	Reference
rs1364043	5-HTR1A	3′UTR	5	63250851	[57], [110]
rs130058	5-HTR1B	5′UTR	6	78173281	[58]
rs1923886	5-HTR2A	intronic	13	47423291	[59]
rs6313	5-HTR2A	exonic(synon)	13	47469940	[48], [61], [111]
rs7997012	5-HTR2_A_	intronic	13	47411985	[62], [63], [112]
rs518147	5-HTR2c	5′UTR	11	113818582	[113]
rs1176744	5-HTR3B	exonic(missense)	11	113803028	[63], [114], [115]
rs10917509	5-HTR6	5′UTR	1	19992066	[64]
rs3788862	MAO A	intronic	X	43517364	[67]
rs2283729	MAO B	intronic	X	43678042	[67]
rs1799836	MAO B	intronic	X	43627999	[98]
rs1042173	SLC6A4	3′UTR	17	28525011	[116]
tri-allelic 5-HTTLPR	SLC6A4	Promoter	17	≈28564374	[31]–[34], [46], [117]
rs2066713	SLC6A4	intronic	17	28551665	[67], [71], [118]
rs4325622	SLC6A4	intronic	17	28526475	[119]
rs3813034	SLC6A4	3′UTR	17	28524804	[72], [120]

The 15 genotyped SNPs were selected from 86 papers investigating genetic association with pain and psychological traits. The included SNPs have previously been significantly (P<0.05) associated with either pain (rs6313, rs3788862, rs2283729, rs1799836, rs2066713 and rs3813034) or personality traits that have been reported to alter pain perception (rs1364043, rs130058, rs1923886, rs6313, rs7997012, rs518147, rs1176744, rs10917509, rs1042173, rs4325622 and rs3813034), or have been reported to be closely associated with pain catastrophizing (e.g. anxiety and neuroticism) (rs1364043, rs6313, rs7997012, rs1176744, rs10917509, rs4325622 and rs3813034). The populations analyzed in the previous studies were Caucasians with the exception of two studies based on Chinese population. SNP genotyping (see Table 1) was carried out using the Sequenom MassARRAY Genotyping platform (Sequenom, San Diego, CA, USA) following the procedure described in [74]. Primer sequences and assay conditions are available from the authors upon request. Only SNPs that preserved the Hardy-Weinberg equilibrium were included in the analysis.

### Pain Catastrophizing

Pain catastrophizing was assessed by means of the Danish version of the PCS, which measures thoughts and feelings when experiencing pain [14]. In agreement with the standard instruction for the PCS, participants were told “we are interested in the thoughts and feelings you have when you are in pain” [14]. The scale has 13 items, and it includes three subscales: “helplessness”, “magnification” and “rumination”. The PCS has previously been used in healthy volunteers with (e.g. [20]) or without pain (e.g. [75]) as well as in chronic pain patients (e.g. [19]). The Danish version of the PCS has previously shown a high reliability (Cronbach’s Alpha:0.956) [19].

### Anxiety

Anxiety was measured with the Danish version of the STAI for adults [76]. The STAI assesses emotional, cognitive and behavioral aspects of anxiety. Form Y is the most frequently used version and has 20 items for assessing trait anxiety (STAI I) and 20 items for assessing state anxiety (STAI II). The Danish version of the STAI has previously shown a reasonable reliability (Cronbach’s Alpha: 0.803) [19].

### Depression

Depression was assessed with the Danish version of the BDI (second edition) [77], which consists of 21 items assessing psychological and physiological aspects of depression. The Danish version of the BDI has previously shown a high reliability (Cronbach’s Alpha: 0.935) [19], [78].

### Statistical Analysis

Tests for deviations from the Hardy-Weinberg equilibrium and allelic association analyses were obtained using the statistical software PLINK (v1.07 http://pngu.mgh.harvard.edu/purcell/plink/). Allelic association analyses were conducted using linear regression applying an additive model (for nonparametric regression method). A significance level of P<0.05 was adopted for all association analyses. Correlations between the three psychological parameters (pain catastrophizing, anxiety and depression) were tested, and they were all significantly correlated (see Table S1). Since the psychological tests are not independent, corrections for multiple comparisons were done only for the 15 SNPs and the tri-allelic polymorphism by Bonferroni test (adjusted α = 0.002).

To detect the power of the sample size to find a true association under the study constraints, the software PGA was used [79]. Power analyses were performed by using 15 independent tests, a false-positive rate of 0.05, a disease prevalence of 0.01, a control to case ratio of two and various settings for the relative risk (1.50, 1.60, 1.70), assuming a codominant model with one degree of freedom, and various settings for the disease allele frequency (0.2, 0.3, 0.4), assuming complete LD between the genotyped marker and the causative SNPs. The power to detect association with a sample size of 379 cases ranged from 29 to 69%.

## Results

Three hundred and eighty participants completed the study. The extracted DNA from one male participant had poor quality and was excluded from genotyping. A total of 379 participants were genotyped. Individuals with a genotyping call rate below 0.7 were excluded (31 participants), thus 348 participants (175 males and 173 females, 24.5±4.5 years, with no age difference between genders) were included in the analysis. Quality control of the SNPs with call rate >0.9 was left for further analyses. No SNPs demonstrated significant deviation from the Hardy-Weinberg equilibrium, and the minor allele frequency (MAF) found was 14%, and all MAFs were >0.1%. Nice cluster plots were observed for all SNPs. Genotyping consistency rate of the tri-allelic polymorphism was 98%.

All associations of polymorphisms with pain catastrophizing (PCS), depression (BDI) and anxiety (STAI) scores are presented in Table 2. The significant associations preserved after Bonferroni corrections for multiple comparisons are described below.

**Table 2:** Minor allele association with personality trait scores.

Polymorphism (dbSNPdatabase number)	gene	MA	MAF [%]	PCS	PCS R	PCS M	PCS H	BDI	STAI I	STAI II
**rs1364043**	5-HTR1A	G	22.82	0.46	−2.77	0.89	−0.15	0.36	0.90	0.53
**rs130058**	5-HTR1B	T	26.8	−0.01	**4.91**	−0.16	−0.10	0.15	−0.18	−0.21
**rs1923886**	5-HTR2A	C	49.5	0.01	−1.04	−1.20	−0.21	0.24	−0.87	−0.97
**rs6313**	5-HTR2A	T	39.0	0.30	1.44	0.46	0.25	0.55	0.48	1.03
**rs7997012**	5-HTR2_A_	A	45.1	−0.08	−0.87	−0.21	−0.03	0.44	−0.21	0.25
**rs518147**	5-HTR2c	G	36.9	−0.12	−1.14	−0.07	0.07	−0.32	−0.10	0.10
**rs1176744**	5-HTR3B	G	33.1	−**0.85**	−4.24	−**2.02**	−**0.38**	−0.55	**2.01**	**1.65**
**rs10917509**	5-HTR6	T	33.9	−0.09	1.06	−0.89	−0.03	−0.25	−0.87	0.12
**rs3788862**	MAO A	A	30.2	0.18	2.07	−0.06	0.11	0.35	−0.05	0.52
**rs1799836**	MAO B	A	48.5	−0.09	0.86	0.06	0.05	−0.13	0.05	−0.60
**rs2283729**	MAO B	A	33.0	0.32	−0.43	−0.11	0.21	0.46	−0.09	0.92
**rs1042173**	SLC6A4	G	44.8	−0.25	−**4.14**	0.10	−0.10	0.76	−0.15	0.19
**rs2066713**	SLC6A4	T	38.1	−0.00	2.02	−0.87	−0.11	−0.35	−0.85	0.11
**rs4325622**	SLC6A4	C	44.7	−0.17	−3.57	0.23	0.20	0.07	0.23	0.17
**rs3813034**	SLC6A4	C	44.9	−0.19	−3.51	0.05	−0.05	0.10	0.05	0.06
**tri-allelic 5-HTTLPR**	SLC6A4	S_A_	19.9	−0.20	−2.07	−0.9	−0.17	0.33	0.9	1.10

Regression coefficient (slopes of the regression) between the minor allele and the psychological trait. MA, minor allele; MAF, observed minor allele frequencies. In bold, significant associations after regression analysis using additive model and preserved after Bonferroni correction for multiple comparisons. PCS, pain catastrophizing scale; PCS R, pain catastrophizing rumination; PCS M, pain catastrophizing magnification; PCS H, pain catastrophizing helplessness. BDI, Beck’s Depression Inventory; STAI, State-Trait Anxiety Inventory I (state) and II (trait).

### Pain Catastrophizing

The G-allele of rs1176744 was negatively correlated with the global score of the PCS (P = 0.001) and with the subcategories of magnification (P = 0.001) and helplessness (P = 0.001) (Table 2). Also, the minor allele of rs1042173 (G-allele) was negatively related with rumination, whereas rs130058 (T-allele) was positively (P = 0.002) correlated with the rumination subscores of the PCS.

#### Anxiety

The minor allele of rs1176744 in the *5-HTR3B* showed a positive correlation with both state anxiety (P = 0.0002) and trait anxiety (P = 0.001) scores (Table 2).

#### Depression

There were no SNPs correlated with BDI scores (Table 2).

## Discussion

Pain catastrophizing, along with anxiety and depression, has been suggested as one of the strongest predictors of pain-related outcomes, showing positive associations with pain report, pain behaviors, analgesic use, length of hospital stay, length of rehabilitation and pain-related disability [1]–[7], [80].

The present study aimed to investigate possible associations between pain catastrophizing, anxiety and depression (psychological variables of relevance for pain perception) and SNPs, mainly in the serotonin (5-HT) pathway. To our knowledge, this is the first study to show an association of rs1176744 in the serotonin receptor 3_B_ (*5-HTR3_B_*) with PCS scores.

The 5-HTR3 is the only serotonin receptor that is a ligand-gated ion channel. The binding of the neurotransmitter serotonin to the 5-HT3 receptor opens the channel, which in turn leads to an excitatory response in neurons. Presynaptic 5-HT3 receptors are considered to mediate or modulate neurotransmitter release (including, e.g., GABA and dopamine) [81], [82], whereas postsynaptic receptors are responsible for the fast excitatory response to 5-HT.

The Rs1176744 polymorphism results in a tyrosine/serine substitution in *5-HTR3_B_*. On a functional level, the tyrosine-allele (Tyr-allele) results in a decreased maximum response to 5-HT due to a sevenfold decrease in single channel mean open time compared to the serine-allele (Ser-allele) [83]–[85]. It has been suggested that the effect of this polymorphism on the 5-HT3_B_ receptor may have an impact on personality traits by affecting serotonin and dopamine signaling [82], [86], which has been predicted by in silico analyses to be “possibly damaging” [86]. In agreement with this finding, the Tyr-allele of rs11767 has previously been found to be overrepresented in patients with major depression [86].

We found a correlation between the T-allele of the SNP (tyrosine in the protein) and higher PCS scores, both the global score and the subscales concerning magnification and helplessness. This is of interest because there are previous reports suggesting a role of spinal 5-HT3 receptors mediating both nociception and antinociception [87]–[93]. Although this diversity of results may arise from differential activation of descending serotonergic systems and by the subfamilies of 5-HT3 receptors studied by individual groups, our results increase the evidence for a role of 5-HT3 in pain modulation. Further studies focusing on the relation between spinal 5-HT3 and pain catastrophizing are needed.

Previous studies have reported an association between alterations in the serotonin system and attentional threat bias [39], [40]. Since there are no studies that have analyzed the possible association between the rs1176744 polymorphism and attentional bias, a further study is required in order to analyze if the correlation between the Tyr-allele and the higher PCS scores is due to an attentional bias toward the pain experience.

Interestingly, we also found that the Tyr-allele was negatively associated with anxiety scores measured with STAI I and STAI II. Although this may sound contradictory at first, it is also in accordance with previous publications that have suggested that 5-HT enhances conditioned anxiety by acting in the forebrain [94]–[96], for review see [97]. Accordingly, it is likely that the Tyr-allele, by diminishing the 5-HT response, decreases the level of anxiety as reflected in lower scores on STAI I and STAI II.

MAO plays an important role in monoamine neurotransmission; however, rs1799836 and rs2283729 in *MAO-B* did not show any correlation with pain catastrophizing or personality trait. Although Dlugos and colleagues [98] found that intronic *MAO-B* SNPs (rs10521432 and rs6651806) were associated with the personality trait of negative emotionality in healthy humans, in agreement with our results, they did not find any association between emotionality and rs1799836. Several lines of evidence indicate that MAO, in particular MAO-A, plays an important role in human behavior and physiology [99]. First, low platelet MAO activity has been linked to vulnerability for depression, suicidality and substance abuse disorders [100]–[103]. Second, MAO inhibitors are used to treat depression [104]–[106]. The SNP rs2283729 was positively related with agreeableness, which reflects a tendency to be friendly and compassionate [107], [108]. This is in agreement with a previous study showing that other polymorphisms affecting MAO have been positively related with agreeableness [109].

Some methodological problems in this study need to be considered. The study did not test if pain catastrophizing *per se*, i.e. independently of anxiety and depression, was related to specific polymorphisms. In so far as there was a correlation between pain catastrophizing, anxiety and depression, it cannot be precluded that anxiety and depression also contributed to the findings. Also, the study only included around 350 participants, so the absence of significant correlations between some of the SNPs and psychological outcomes could be the result of the low sample size. In addition, due to the low number of participants, the effect of possible haplotypes had to be excluded from the study in order to avoid underpowered groups. It is also important to remember that participants with a diagnosis of psychological disorder (e.g. depression) were excluded from participation in the study; therefore it is possible that this bias is reflected in the absence of a significant correlation between genetic variations and psychological outcomes such as depression.

Finally, the study was carried out only using participants born in Scandinavia and with Scandinavian descent. Due to the possible differences in allele frequency in other ethnicities, correlations deviating from the ones observed here could be found in samples with different ethnicity. Thus, the results presented in this article should be replicated in larger samples including groups with different ethnic origin.

In this study, we found an association between the SNP rs1176744 in the serotonin receptor 3_B_ (5-HTR3_B_) and PCS scores, suggesting a role of the serotonin pathway in pain catastrophizing. Further studies are needed to investigate the molecular process behind the interaction between serotonin and pain catastrophizing; in addition it should be examined whether the interaction of polymorphisms in the serotonin pathways enhance or reduce this association.

## Supporting Information

Table S1 **Regression coefficient (slopes) of the correlation between psychological traits.** *P<0.05: **P<0.005; ***P<0.001. PCS, pain catastrophizing scale; BDI, Beck’s Depression Inventory; STAI, State-Trait Anxiety Inventory I (state) and II (trait).
(DOCX)
