# Supplementary material for: Polymorphism in Serotonin Receptor 3B Is Associated with Pain Catastrophizing
Source: PLoS One. 2013 Nov 11;8(11):e78889. doi: 10.1371/journal.pone.0078889 (PMC3823944; doi:10.1371/journal.pone.0078889)
Supplement: Table S1 — Regression coefficient (slopes) of the correlation between psychological traits. *P<0.05: **P<0.005; ***P<0.001. PCS, pain catastrophizing scale; BDI, Beck’s Depression Inventory; STAI, State-Trait Anxiety Inventory I (state) and II (trait). (DOCX) [file pone.0078889.s001.docx]

**Supplementary table1**

|  | **PCS** | **BDI** | **STAI-I** |
| --- | --- | --- | --- |
| **BDI** | 0.33* |  |  |
| **STAI-I** | 0.32** | 0.61** |  |
| **STAI-II** | 0.36* | 0.71** | 0.69*** |
